# Microbiome features associated with performance measures in athletic and non-athletic individuals: A case-control study

**DOI:** 10.1371/journal.pone.0297858

**Published:** 2024-02-21

**Authors:** Kinga Humińska-Lisowska, Kinga Zielińska, Jan Mieszkowski, Monika Michałowska-Sawczyn, Paweł Cięszczyk, Paweł P Łabaj, Bartosz Wasąg, Barbara Frączek, Anna Grzywacz, Andrzej Kochanowicz, Tomasz Kosciolek

**Affiliations:** 1 Faculty of Physical Culture, Gdansk University of Physical Education and Sport, Gdansk, Poland; 2 Malopolska Centre of Biotechnology, Jagiellonian University, Cracow, Poland; 3 Faculty of Health Sciences, University of Lomza, Lomza, Poland; 4 Department of Biology and Medical Genetics, Medical University of Gdansk, Gdansk, Poland; 5 Department of Sports Medicine and Human Nutrition, Institute of Biomedical Sciences, University School of Physical Education, Cracow, Poland; 6 Department of Data Science and Engineering, Silesian University of Technology, Gliwice, Poland; Dynamical Business & Science Society - DBSS International SAS, COLOMBIA

## Abstract

The influence of human gut microbiota on health and disease is now commonly appreciated. Therefore, it is not surprising that microbiome research has found interest in the sports community, hoping to improve health and optimize performance. Comparative studies found new species or pathways that were more enriched in elites than sedentary controls. In addition, sport-specific and performance-level-specific microbiome features have been identified. However, the results remain inconclusive and indicate the need for further assessment. In this case-control study, we tested two athletic populations (i.e. strength athletes, endurance athletes) and a non-athletic, but physically active, control group across two acute exercise bouts, separated by a 2-week period, that measured explosive and high intensity fitness level (repeated 30-s all-out Wingate test (WT)) and cardiorespiratory fitness level (Bruce Treadmill Test). While we did not identify any group differences in alpha and beta diversity or significant differential abundance of microbiome components at baseline, one-third of the species identified were unique to each group. Longitudinal sample (pre- and post-exercise) analysis revealed an abundance of *Alistipes communis* in the strength group during the WT and 88 species with notable between-group differences during the Bruce Test. SparCC recognized *Bifidobacterium longum* and *Bifidobacterium adolescentis*, short-chain fatty acid producers with probiotic properties, species strongly associated with VO_2_max. Ultimately, we identified several taxa with different baseline abundances and longitudinal changes when comparing individuals based on their VO_2_max, average power, and maximal power parameters. Our results confirmed that the health status of individuals are consistent with assumptions about microbiome health. Furthermore, our findings indicate that microbiome features are associated with better performance previously identified in elite athletes.

## Introduction

The human microbiota refers to 10–100 trillion symbiotic microbial cells, mainly human gut bacteria, and the set of genes they possess is termed the human microbiome [1]. Thanks to initiatives such as the Human Microbiome Project [2] and the American Gut Project [3], interest in the functions of the microbiome and its role in maintaining human health is growing [4]. This vast and dynamic microbial ecosystem plays an integral role in various aspects of host physiology, including digestion, metabolism, and immune modulation [5]. One of the remarkable attributes of the gut microbiome is its capacity to produce bioactive metabolites, including short-chain fatty acids (SCFAs) [6]. SCFAs, such as acetate, propionate, and butyrate, are key metabolic products of microbial fermentation in the colon. They contribute to the regulation of gut homeostasis, modulate the immune system, and even influence brain function via the gut-brain axis [7]. Therefore, the role of the gut microbiota in extracting energy from our diet and contributing to nutrient absorption and storage cannot be neglected.

The human microbiome is influenced by a wide range of host factors, such as sleep [8], diet [9], and age [10], including other environmental determinants (host genome, stress, illness, and drugs) [11]. Any disruption to the microbiome homeostatic state, collectively known as dysbiosis, is thought to have a number of consequences and may offer a mechanistic link to immunoregulatory disorders, including allergies, digestive system diseases, autoimmune diseases, metabolic diseases, CNS-related diseases, depression and others [12]. However, not all changes in microbiome composition are negative. The human body’s response to external stimuli is highly adaptive and necessary for inducing positive long-term health gains. One such factor affecting human bodily function via microbiome changes is physical activity.

The beneficial effects of a physically-active lifestyle are well established. Physical exercise may induce both acute and chronic alterations in the microbiome, and is widely agreed to be an adaptive stimulus for promoting positive health outcomes [13]. Alterations in the health-associated microbiome are also known. Research has shown that microbiome activity is linked to sports performance [14], and could eventually guide the optimization of each athlete’s abilities by changing microbiome composition for targeted performance improvements. Apart from sports applications, this could have practical implications in treating more general health disorders, such as obesity and cardiovascular diseases [15].

Some studies have suggested that athletic gut microbiota has higher levels of alpha diversity and health-supporting gut bacteria [16]. While studies remain inconsistent, species such as *Faecalibacterium prausnitzii*, *Roseburia hominis*, *Akkermansia muciniphila* and *Eubacterium rectale* are generally cited as having a positive influence on health and performance [17]. Athletes also tend to have an enrichment of SCFAs modulating substrate metabolism at various organs [18]. Bacterial species that affect SCFA production or breakdown, such as *Veillonella atypica*, have been shown to positively influence marathon running time [19]. Furthermore, athletes often express several enriched pathways compared to sedentary individuals, including amino acid and antibiotic biosynthesis, and carbohydrate metabolism [20].

The features of a healthy athletic microbiome are not universal, but are highly dependent on individuals and the specific characteristics of their chosen sport. For example, endurance-type exercises are mainly associated with cardiorespiratory components of fitness, leading to specific metabolic adaptations. Endurance training is known to increase the production of SCFAs; this being particularly advantageous during prolonged efforts, as they contribute to energy maintenance. Additionally, endurance-oriented physical activity has been consistently linked to an abundance of microbiota species known for their anti-inflammatory properties [13]. Conversely, strength-based sports emphasize enhanced protein utilization to support muscle development and performance. It was observed that enhanced use of protein in strength sports decreased the occurrence of SCFA-producing bacteria, redundant in such kinds of exercise–and replaced them with species associated with strength training [21]. These observations underscore the intricate relationship between different forms of physical activity and gut microbiota.

Comparative studies are, however, difficult to interpret due to the testing of differences between elite athletes (healthy or not) and sedentary, often unhealthy, individuals. Subsequently, it is not entirely clear which microbiome features are strictly linked to better health or optimal sports performance. Furthermore, published reports comparing different athletic populations are usually inconclusive [22], referenced against different control groups, and involve determination of ambiguous athletic microbiome markers.

This study sought to evaluate gut microbiota and its response to acute exercise interventions performed at maximal intensity. Since post-exercise microbiome responses are contingent on the type of discipline practiced, we tested both endurance and strength athletes. A control group were also recruited for comparative purposes. In order to focus on the performance-associated microbiome characteristics, only healthy individuals were recruited in this study. Specifically, the athletes were physically active, but not training or competing at a professional level. To gain insights into gut microbiota alterations related to different exercise modalities [23], two exercise interventions were performed to assess; (1) an explosive or high intensity component of fitness and (2) a cardiorespiratory fitness component. As a result, we were able to eliminate the “health factor” and focus solely on pre-exercise and post-exercise microbiome alterations across three groups (strength athletes, endurance athletes, controls), each performing two different exercise modalities (anaerobic and aerobic).

## Materials and methods

### Participants

To determine the appropriate sample size, a power analysis of the interactions between effects was performed using GPower (version 3.1.9.2). The minimal total sample size for a medium effect size, at a power of 0.8 and a significance level of 0.05, was calculated at 15 subjects each group. Thus, a total of 52 men were recruited for this study and assigned to one of three groups: endurance athletes (n = 15), strength athletes (n = 16), and a control group (n = 21). The endurance athletes self-reported a minimum of 5 years of training, focusing on race walking, long-distance running (marathon, half marathon, ultra-running), or ski running. The strength athletes reported a minimum of 5 years of training in weightlifting, powerlifting, and/or bodybuilding activities. For the control group, only men who did not participate in any organized training, but were physically active (participating in amateur team-sport activities, occasional gym training and swimming), were included. Exclusion and inclusion criteria are presented in Table 1. The subjects provided written informed consent before enrolment. They were informed about the study procedures; however, they were not aware of the rationale and study aims, and therefore, were naive about the main scope of this work.

**Table 1:** Eligibility criteria.

**Inclusion criteria**	1. Aged from 19 to 24 years or older.
	2. Experience in sports discipline (for the endurance or strength population) or any professional sports performance.
	3. Good health status.
	4. No additional drug intake, alcohol consumption, or smoking.
	5. No additional probiotic and antioxidant supplementation.
	6. For the endurance population: at least 5 years of training and starting (preceding the study) in endurance disciplines such as race walking, long-distance running (marathon, half marathon, ultrarunning), or ski running.
	7. For the strength population: at least 5 years of training and starting (preceding the study) in strength disciplines such as weightlifting, powerlifting, and bodybuilding.
	8. For the control population: no participation in any structured professional training, but recreational participation in sports such as running, swimming, and team sports (on average, 3–4 times a week for a duration of 45 minutes) 3–4
**Exclusion criteria**	1. Physically or mentally compromised individuals (currently treated for a psychiatric disorder or alcohol or substance abuse), unwilling or unable to comply with study evaluations
	2. Comorbidities causing severe inflammation: Addison’s disease, allergy, asthma, celiac disease, psoriasis, Raynaud’s disease, rheumatoid arthritis, systemic lupus erythematosus, type 1 diabetes, and others.
	3. Gastrointestinal tract problems (irritation of the gastric mucosa, vomiting, frequent abdominal pain, and dyspeptic disorders of unknown etiology).
	4. Gastrointestinal diseases, ex. irritable bowel syndrome (IBS), Infectious colitis, Crohn’s disease, Ulcerative colitis, gallstones, fecal incontinence, lactose intolerance, Hirschsprung disease, abdominal adhesions, Barrett’s esophagus, appendicitis, indigestion (dyspepsia), intestinal pseudo-obstruction, pancreatitis, short bowel syndrome, Whipple’s disease, Zollinger-Ellison syndrome, malabsorption syndromes and hepatitis and other.
	5. Use of any antibiotics, synthetic (nonantibiotic) antimicrobial agents, or any anti-inflammatory drugs in a period of 6 months before the start of the study
	6. Taking laxatives or substances affecting the gastrointestinal tract (prokinetics etc.)

None of the participants had any history of known diseases or reported taking medication (antibiotics or probiotics) for any illness six months before this experiment. Moreover, they did not have direct contact with people with advanced bacterial, viral, or fungal diseases. The study protocols were approved by the Bioethics Committee for Clinical Research of the Regional Medical Society in Gdansk (KB-27/18) and conducted in accordance with the Declaration of Helsinki. All participants were informed of the possibility of withdrawing from the study at any time, for any reason and without consequences. To ensure anonymity and privacy, all personal data were encoded upon recruitment and only information regarding the sport disciplines were accessible. Five individuals were excluded from this study, due to illness or injury.

### Experimental overview

This is an interventional, case-control study with repeated measures [24]. Each group were assessed for fecal microbiome DNA composition before and after two different exercise tests; (1) a repeated 30-s all-out Wingate test (WT) and (2) the Bruce Treadmill Test. Fitness testing was performed at the Gdansk University of Physical Education (Gdansk, Poland) in October 2018. A 14-day break separated the Wingate and Bruce Treadmill Tests. During this period, each participant was requested to engage in their usual physical activities and training routines. A professional physician examined all the volunteers before and after each test. A week before testing, all participants attended a familiarization session to ensure that they were accustomed with the testing equipment and study procedures.

Before qualifying for this study, all participants completed a questionnaire on dietary preferences and physical activity to determine group assignment (i.e. endurance, strength, or controls). The participants were instructed to maintain their everyday diet, but were asked to avoid caffeine, alcohol consumption, and tobacco smoking during the week preceding the testing date, up to the last sample collection. Food was not consumed during testing and water was available *ad libitum*.

At the recruitment stage, the dietary intake of participants was also assessed. Results of this analysis, regarding the frequency of food consumption and general eating habits, were used to identify nutritional irregularities and to develop individual suggestions for dietary control. In particular, recommendations were presented regarding food intake (e.g. macronutrient needs), whilst adhering to the principles of proper composition, size of meals and hydration. To ensure appropriate nutritional conditions were met, each participant received sample menus based on their nutritional preferences and lifestyle needs. Dietary recommendations were given for at least two weeks before the start of each exercise test until the last stool collection. On the day before each exercise test and on the day of the testing, participants received meals at Gdansk University of Physical Education, established by the dietitian according to individual energy expenditure.

### Measurement of high-intensity fitness: Repeated lower body 30-s all-out Wingate test

The WT was conducted on a friction-loaded cycle ergometer (Monark 894E, Peak Bike from Sweden). Seat height was adjusted for the height and limb length of each participant, so that the final knee angle was approximately 170°–175°. The participants’ feet were held firmly in place (by pedal clips) to ensure complete contact with the pedals. Before exercise testing, each individual completed a standardized warm-up on the cycle ergometer (5 min at 60 rpm, 1W/kg). For the main exercise, each participant was required to pedal with maximum effort for 30 s against a fixed resistive load of 75 g/kg of total body mass, as recommended by Bar-Or (1987) [25]. After the first test, participants had a 30 s break, and the WT was repeated with maximum verbal encouragement [25]. Several performance metrics were evaluated during the WT: peak power (W) and relative peak power (W/kg) were calculated as the highest single point of power output (recorded at 0.2 s intervals); mean power (W) and relative mean power (W/kg) were computed as average power output during each 30 s bout.

### Measurement of cardiorespiratory fitness: Bruce treadmill test

The Bruce Treadmill Test was conducted two weeks after the WT (in October 2018). The participants completed the Bruce Protocol on an electric treadmill ergometer (H/P/Cosmos, Germany). Following a standardized warm-up (5 min with 60% HR max), each participant ran on the treadmill with increasing loads, including running velocity and treadmill inclination. Specifically, they performed 10 test stages with an incremental rise in running difficulty, starting with a 10% incline and running at 2.7 km/h (level 1) and ending with a 28% incline and running at 12.07 km/h (level 10). The test was stopped when the subject could not continue owing to fatigue or other conditions [26, 27]. During the test, participants wore face masks connected to a pulmonary gas exchange analyzer (Quark CPET, Cosmed, Italy) and maximal oxygen uptake was measured.

### Sample collection

All participants collected fresh stool samples before and after each exercise intervention at three time points: T0 (W0 or B0), in the morning after an overnight fast of ~8 hours before the exercise test; T1 (W1 or B1), on the same day after the exercise test; and T2 (W2 or B2), the next morning on an empty stomach. The samples were collected in stool containers, immediately placed in thermal bags (with cooling inserts) and stored in a fridge for a short period. The samples were delivered to the laboratory within a maximum of a few hours, and immediately snap-frozen in a -80°C freezer.

### Sample preparation

#### DNA isolation, quantitation and quantification

The frozen stool samples were thawed on ice. DNA was extracted from 200 mg of stool sample using a modified NucleoSpin^®^ DNA Stool kit (Macherey-Nagel, Germany) via physical, mechanical, and chemical lysis (in March—May 2020). Mechanical lysis using a Bead Ruptor Elite (Omni International, US) was used to improve lysis. The following homogenization protocol was used: 15 s homogenization with 6 ms speed × 5 times with a 2 min break after each 15 s cycle. The concentration and purity of total DNA isolates in each sample were measured spectrophotometrically using a NanoDrop (Thermo Scientific, USA) at wavelengths of A260 and A280. The degree of DNA degradation and potential contamination were analyzed using 1% agarose gels. Good quality and quantity DNA samples were snap-frozen, and stored at -80°C. Prior to library preparation, the quantity of DNA was evaluated using a Qubit fluorometer and Qubit DNA BR Assay Kit in a Qubit^®^ 3.0 Fluorometer (Thermo Fisher Scientific, USA). The final DNA concentration was standardized directly prior to library preparation.

#### Library preparation and sequencing

The library preparation procedure (performed in March–May 2022) followed the KAPA HyperPlus protocol (Roche, Basel, Switzerland). Stool-extracted DNA (100 ng of stool-extracted DNA) was used for the library preparation. Eighteen minutes of fragmentation at 37°C was applied to the library’s 300–500 bp average fragment size. Indexed adapter ligation was performed using KAPA Universal dual-indexed adapters (Roche) at a concentration of 15 μM. To increase efficiency, adapter ligation was performed at 20°C for 1h. After ligation, the libraries were purified using a 0.8X KAPA Pure Bead (Roche, Switzerland) cleanup to remove unincorporated adapters. Six PCR cycles were performed during library amplification. The following cycling conditions were applied: 98°C for 45 s; six cycles of 98°C for 15 s, 60°C for 30 s, and 72°C for 30 s; final elongation at 72°C for 1 min. Amplified fragments with adapters and tags were purified using KAPA Pure beads (Roche, Switzerland) at a 1:1 concentration.

Purified libraries were stored for up to 2 weeks at − 20°C until sequencing. The quality of the libraries and fragment distribution were analyzed using a 4150 TapeStation System and D1000 reagents (Agilent Technologies, USA). Quality control of the purified libraries was performed for up to two weeks before the sequencing run. Prior to sequencing in June–August 2022, all libraries were thawed on ice and normalized using the Qubit DNA HS Assay Kit (Thermo Fisher Scientific, USA) to a final concentration of 20 nM and mixed in equimolar concentrations to ensure an even representation of reads per sample. The libraries were sequenced on an Illumina NovaSeq sequencer (Novogene, Cambridge, MA, USA), and 150 bp paired-end reads were generated. Approximately 22 million 150 bp paired-end reads were generated per library.

### Statistics and bioinformatics analysis

Raw sequencing reads were preprocessed using Trim Galore! 0.6.10 [28]. Taxonomic profiles were calculated using Metaphlan4 [29], while functional and gene profiles were obtained using Humann 3.6 [30]. Between-group comparisons at all time points were performed using Qiime2 [31]: alpha and beta diversity, ANCOM, and LEfSe [32]. The Kruskal-Wallis test was used to compare alpha diversity, and PERMANOVA to compare beta diversity. Other statistical tests were based on the default output of tools applied, and results were considered to be statistically significant if *p* < 0.05 (unless stated otherwise). Longitudinal analysis was performed using the Qiime2 longitudinal plugin. Due to a limited number of samples collected prior to the WT test (n = 6) and a two-week break between the WT and Bruce tests, comparative analysis was conducted between W0 and B0 only. We confirmed the validity of using B0 instead of W0 samples by showing that, according to the Bray-Curtis dissimilarity index, the few baseline WT samples were not further from non-baseline WT or Bruce samples than the Bruce control samples. This index considers the taxonomic composition of samples and grades samples on a continuous scale according to species similarity (from 0 = same, up to 1 = none). Correlations between microbiome features and metadata were calculated using MaAsLin [33]. Results are presented visually using custom-made Python scripts. Authors who analyzed the study data had no access to information that could identify individual participants.

## Results

### Group comparisons at baseline

**Fig 1:**
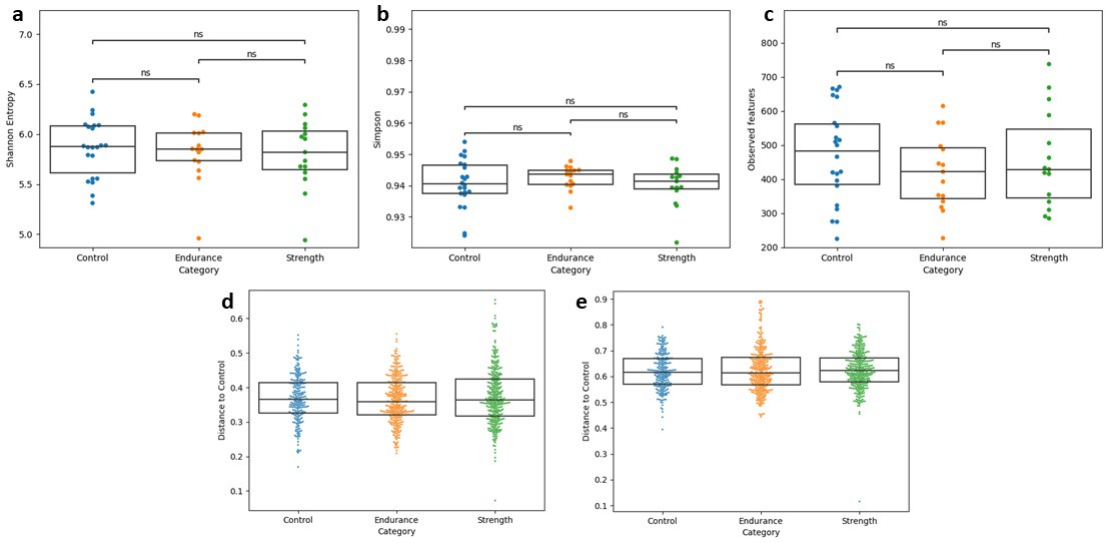
Comparison of alpha and beta diversity of the control, endurance and strength groups. **a)** Shannon entropy. **b)** Simpson index. **c)** Observed features. **d)** Bray-Curtis distance. **e)** Jaccard distance.

The analysis of baseline samples revealed no significant differences in alpha and beta diversity between the control, strength and endurance groups (Fig 1). More detailed alpha diversity (Shannon entropy, Simpson and Observed features) and beta diversity (Bray-Curtis and Jaccard) statistics are shown in S1 and S2 Tables.

**Fig 2:**
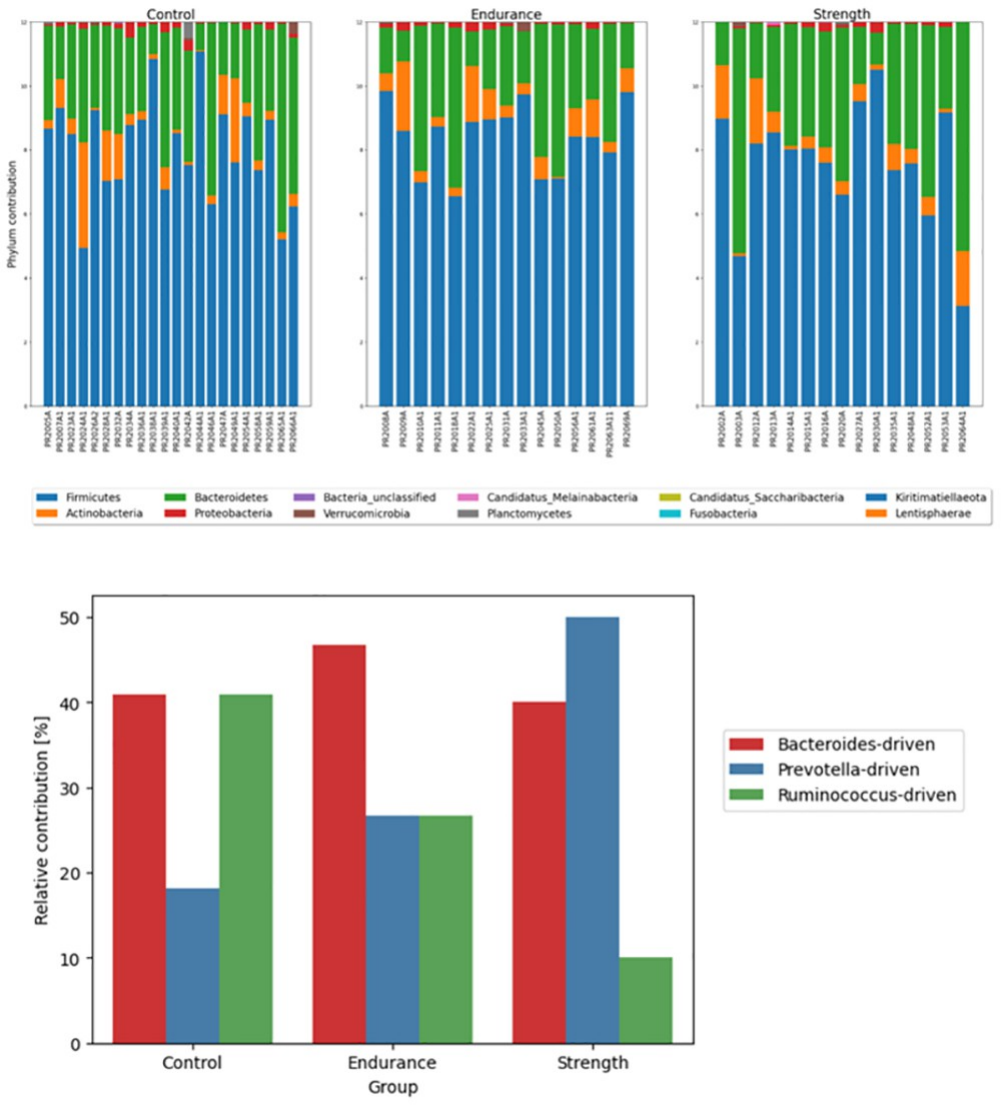
**A**. Phyla contributions in every sample, grouped by Control, Endurance and Strength. **B**. Enterotype contributions to Control, Endurance and Strength groups.

There were no observable differences in the proportions of phyla among the control, strength and endurance groups (Fig 2). The most abundant genus was *Firmicutes*, followed by *Bacteroidetes* and *Actinobacteria* (average relative abundance of 66.7%, 25.9%, and 5.9%, respectively). *Bacteroidetes* contributed to approximately half of the population in the three samples (one control and two strength), whereas *Firmicutes* constituted the majority in most individuals. We also investigated enterotypes, which were previously defined as microbiota profile types [34]. The *Prevotella*-driven enterotype is associated with high fat and protein intake in strength athletes, while the *Bacteroides*-driven enterotype is linked to simple carbohydrates commonly ingested by endurance athletes [14]. *Bacteroides*-driven and *Ruminococcus*-driven enterotypes were equally prevalent in the control group (40.9% each). *Bacteroides*-driven enterotype was dominant in the endurance group (46.7%), whereas *Prevotella*-driven enterotype was dominant in the strength group (50.0%). In this work, participants’ diets were standardized depending on energy expenditure and remained isocaloric throughout the study. However, due to a lack of detailed nutritional analysis, the extent of diet influence on enterotype formation could not be determined.

**Fig 3:**
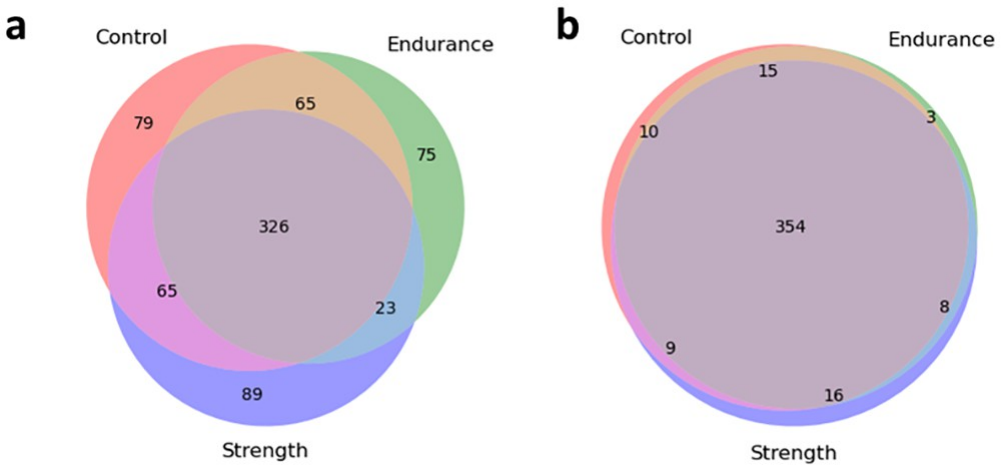
Overlap of a) species and b) functions identified in each group at baseline.

The intersection of species and functions in each group is presented in Fig 3. While nearly 34% of all species identified were unique to either group, only 7% of the functions occurred in a single group. An equal number of species were shared between control and endurance and control and strength, whereas three times fewer species were shared between endurance and strength. It was particularly interesting to see an enrichment of indigestible carbohydrate degrading bacteria [35] (*Blautia genus*) in the endurance group, suggesting performance-specific adaptations. We also observed a presence of *Christensenella minuta* (a potential therapeutic target associated with the health of the gut) [36] and *Gordonibacter urolithinfaciens* (one of the few species able to convert ellagic acid to urolithins) [37]. Furthermore, a number of probiotic taxa (*Lactobacillus kalixensis* [38], and *Leuconostoc pseudomesenteroides* [39], *Monoglobus pectinilyticus* [40]) with yet poorly defined mechanisms of action were found in the trained, but not untrained, individuals. The controls shared more pathways with endurance athletes, whereas a similar number of functions were shared between controls and strength athletes, and the strength and endurance groups. The complete list of species and functions unique to each group are shown in S3 Table.

### Group comparisons in response to exercise

Initial testing involved a cross-sectional comparison of the strength, endurance and control groups at each time point (W1, W2, B0, B1, and B2). Differential abundance analysis with ANCOM showed no significant taxonomic, functional, or gene differences between the control, strength and endurance groups. The results produced by LEfSe, which has less stringent thresholds than ANCOM [41], also did not reveal many between-group variations (Table 2). *Blautia sp AF19 10LB* was identified as being significantly more abundant in endurance athletes versus controls and strength athletes at most time points, though its implications for human physiology are not yet known, but it has shown a series of potential probiotic properties [42]. No other species were identified at multiple time points within a single group.

**Table 2:** Species identified as specific per group after being identified as significantly more abundant in the group of interest in comparison to the other two groups by LEfSe.

• Timepoint	• Control-specific	• Endurance-specific	• Strength-specific
• B0	• -	• *Blautia sp AF19 10LB* • *Dorea longicatena*	• *Catenibacterium sp AM22 15* • *Clostridium phooceensis*
• W1	• *Bacteroides faecis*	• *Fusicatenibacter saccharivorans*	• *Ruminococcus sp AF41 9* • *GGB3571 SGB4778* • *Rothia mucilaginosa* • *Coprococcus catus*
• W2	• *Phocaeicola massiliensis*	• *Blautia sp AF19 10LB*	• *GGB2977 SGB3959*
• B1	• -	• *Blautia sp AF19 10LB* • *Lacticaseibacillus paracasei*	• *Clostridiales bacterium*
• B2	• -	• *Blautia sp AF19 10LB*	• *Blautia sp MSK 20 85*

B0: in the morning before the Bruce Treadmill Test, B1: the same day after the Bruce Treadmill Test, B2: morning after the Bruce Treadmill Test on an empty stomach, W1: the same day after the repeated lower body 30-s all-out Wingate test, W2: morning fasting after the WT on an empty stomach

LEfSe analysis of functional profiles indicated that one pathway (PWY 5695: inosine 5’-phosphate degradation) was significantly more enriched in endurance versus control groups, but not in strength athletes. Six pathways were identified in endurance versus strength, but not in the control group. These included PWY 7229: superpathway of *de novo* adenosine nucleotide biosynthesis, PWY 841: superpathway of *de novo* purine nucleotide biosynthesis, PWY 7400: L-arginine biosynthesis, PWY 6126: superpathway of *de novo* adenosine nucleotide biosynthesis, PWY 6703: preQ0 biosynthesis, and PWY 7228: superpathway of *de novo* guanosine nucleotide biosynthesis. Interestingly, the same pathways were reported as being enriched in elite Irish athletes performing high static component sports, such as triathlon or cycling [43]. The six pathways enriched in endurance versus strength athletes, but not controls, warrants particularly attention, as they could indicate mechanisms somehow compromised by strength training. Indeed, being linked to NO synthesis (resulting in improved mitochondrial biogenesis and gas exchange) and influencing the TCA cycle, they appeared to be more aerobic-oriented, though the effects of amino acids (e.g. L-arginine) had been positively linked to both aerobic and cardiorespiratory fitness interventions [44]. No significantly enriched genes were identified in this study.

**Fig 4:**
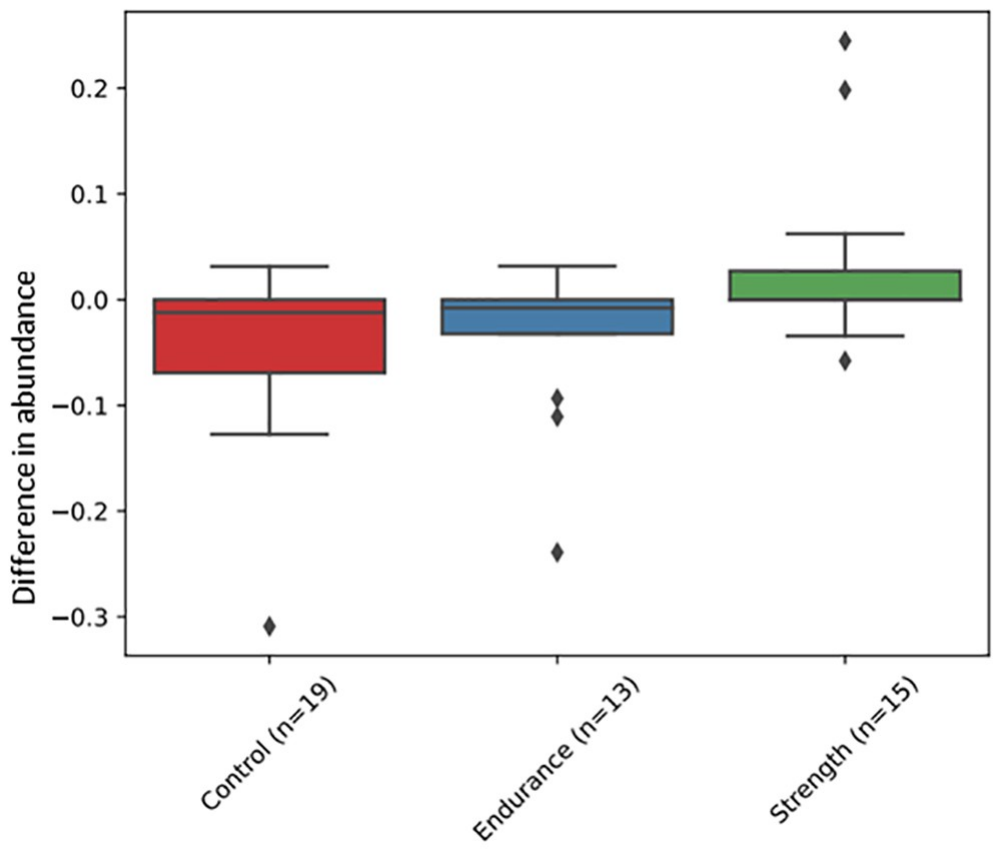
The difference in abundance of *Alistipes communis* between baseline (in the morning fasting before the WT) and W2 (the day after the WT on an empty stomach) per category.

Longitudinal analysis of alpha (Shannon entropy) and beta (Bray-Curtis) diversities revealed no significant differences between any pair of the Bruce intervention timepoints (T0-T1, T0-T2, T1-T2), within or between any of the groups. Due to the lack of baseline WT samples, longitudinal analysis of the explosive and high intensity components of fitness interventions could not be performed. However, as baseline Bruce and WT samples were not significantly distant from one another (see Methods section), we decided to substitute the missing WT samples with the baseline Bruce samples to estimate changes with the former intervention. Interestingly, *Alistipes communis*, yet underexplored regarding its implications for human health [45], but positively correlated with resistance training [46], changed markedly across the WT. Despite a slight net decrease across all individuals combined (average net change = − 0.0004), it appeared to increase steadily in the strength group (Fig 4). Statistical analysis confirmed a significant decrease in *Alistipes communis* in the control (Wilcoxon signed-rank test, FDR P-value = 0.03) and endurance (Wilcoxon signed-rank test, FDR P-value = 0.04) groups, between the baseline and the second time point post-WT. Furthermore, there was a statistically significant difference between the strength and control groups (Mann-Whitney U test, FDR P-value < 0.02), and the strength and endurance athletes (Mann-Whitney U test, FDR P-value < 0.02).

88 species were identified throughout the Bruce Treadmill Test. *Blautia wexlerae*, *Bacteroides ovatus* and *Bacteroides thetaiotaomicron* displayed the greatest positive net average changes (average net changes of 0.007, 0.002, and 0.002, respectively). *Blautia wexlerae* is a short-chain fatty acid producer [21], while *Bacteroidetes* have a wide range of genes responsible for carbohydrate metabolism [47]. The most significant net negative change was experienced by *Fusicatenibacter saccharivorans* (average net change of -0.009); a short-chain fatty acid producer and a core component of the human gut microbiota [14].

Several species showed different responses across groups. *Anaeroburyticium hallii*, a strict anaerobe that utilizes glucose and is involved in acetate and lactate metabolism to butyrate and hydrogen [48], initially increased at B1 and subsequently decreased at B2 in controls, but a reverse trend emerged in endurance and strength athletes. *Ruminococcus bromii* showed an initial decrease followed by an increase at B2 in strength and endurance athletes, but no change for controls. This species is recognized as a short-chain fatty acid producer with anti-inflammatory properties [49]. *Bacteroides xylanisolvens*, known for its probiotic properties [50], displayed a gradual increase in all groups at first, but decreased strongly in the controls at B2, while it continued to increase in the strength and endurance athletes. Finally, *Roseburia hominis* increased in the strength and endurance groups, but not the controls. This short-chain fatty acid producer, involved in maintaining homeostasis in the gut [51], has been shown to increase in abundance in well-trained rowers throughout an ultra-endurance event [52]. Another species that increased in the rowing event was *Dorea longicatena*, which also showed expansion in the endurance and control groups at B1 and strength athletes at B2.

### Species linked to different aspects of sports performance

Despite the interesting trends identified by Qiime2 in the longitudinal analysis, each group contained substantial variation. Therefore, it is not appropriate to define these findings as definitive group-describing characteristics. Because these differences were not as significant as expected, we considered actual physical variation between and within groups as a confounder, and whether it could explain the diversity in microbiome shifts. The features considered included general fitness (fitness score), cardiorespiratory fitness (VO_2_max measured during the Bruce test), explosive and high intensity components of fitness(maximum and average power per kg of body weight during the WT), and the ability to maintain power (fraction of max and average power per kg of body weight maintained in the second part of the WT). Analysis of the WT and Bruce intervention results did not real any visible between-group differences regarding these fitness parameters (S1 Fig).

Significant within-group variation was present, and some even resembled bimodal distributions (VO_2_max in endurance, average power per body weight for strength). This could explain the wide range of longitudinal taxonomic trends across individuals from the same group, as described in earlier sections. No visible correlations of enterotypes with fitness parameter values were observed, apart from two endurance and strength individuals with seemingly better abilities to maintain both average and maximum power throughout the WT, who were *Ruminococcus* dominant enterotypes.

**Fig 5:**
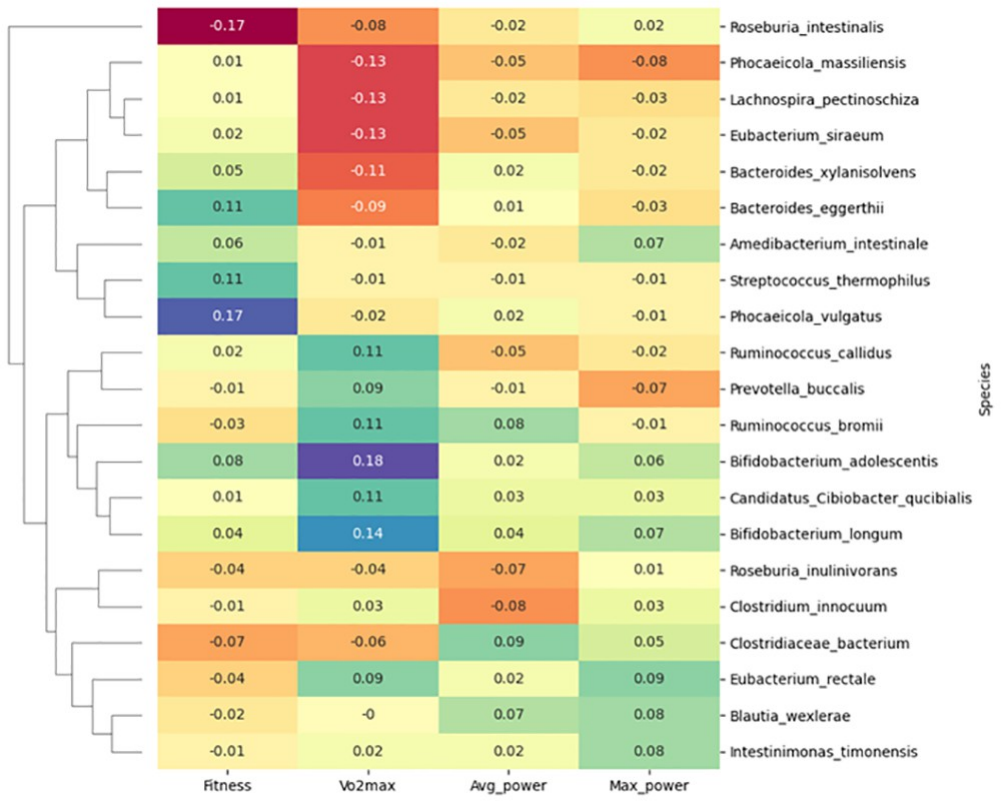
Top SparCC correlations of species with fitness parameters.

MaAsLin was used to search for linear correlations between the microbiota at baseline and parameter values. The lack of significant findings could be explained by either an absence of interactions or more complex connections of bacterial species with sports performance. When we applied the SparCC method, a range of microbial, functional, and gene associations with the fitness score, VO_2_max, average power, and maximal power emerged (Fig 5), suggesting the latter. The species with the highest correlation values were associated with VO_2_max. These included *Bifidobacterium adolescentis* (r = 0.18) and *Bifidobacterium longum* (r = 0.14), which are popular probiotics with positive effects on athletic performance [53, 54]. We also found strong associations between *Phocaeicola vulgatus* and *Roseburia intestinalis* and fitness scores (r values of 0.17 and -0.17, respectively). Although the function of *Phocaeicola vulgatus* remains ambiguous [55], *Roseburia intestinalis* is known to prevent inflammation and maintain homeostasis [56]. The correlations were specific to a few parameters, and no universal set of bacteria influencing all parameters could be identified in the same way. A complete list of the top species correlating with each fitness parameter is shown in S5 Table.

**Fig 6:**
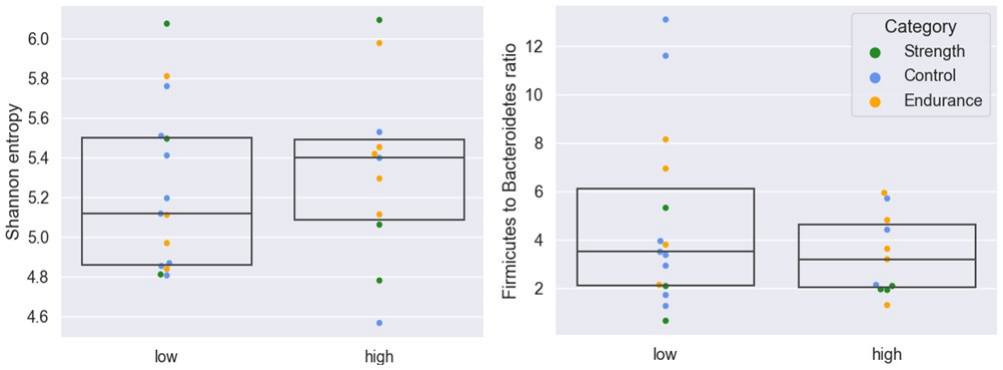
Comparison of high and low VO_2_max groups.

When we investigated the top SparCC correlations using functions instead of taxa (r > = 0.6), only *PWY-7238*: *sucrose biosynthesis II* correlated with two different parameters (r ≥ 0.6, fitness score and average power). However, the direction of the correlations opposed each other (positive for fitness score and negative for average power), and connections with different species were noted (*Bifidobacterium adolescentis* for fitness score and *Blautia wexlerae* for average power). The functions with the highest correlations included inosine 5’-phosphate degradation and phosphopantothenate biosynthesis for fitness score, purine ribonucleoside degradation and chorismite biosynthesis for VO_2_max, sucrose biosynthesis and L-arginine biosynthesis for average power, and molybdopterin biosynthesis and fatty acid biosynthesis for maximum power. Interestingly, some species were associated with multiple parameters, but the species shown in Fig 6 were not among them. *Fusicatenibacter saccharivorans* was associated with functions that correlate with fitness score, VO_2_max, and maximum power; *Blautia obeum* with VO_2_max and maximum power; *Faecalibacterium prausnitzii* with VO_2_max and average power; and *Ruminococcus torques* with fitness score and VO_2_max.

Analysis of gene correlations with fitness parameters revealed a few pathways correlated with multiple parameters, but usually in opposite directions, and associated with different species. RXN-9536 (3-oxo-meristoyl-[acyl-carrier protein] reductase), however, was associated with the fitness score and average power and positively correlated with *Blautia obeum* in both cases.

In addition to the microbiota correlations with parameter values, differences between the relatively high and low parameter groups were investigated. Average power per kg and VO_2_max were selected as the best representative measures of the Wingate and Bruce tests, respectively. Naturally, because all participants were physically active, fit and healthy, both average power and VO_2_max values were substantially higher than in the general population; ranked as”very good" and "excellent” according to VO_2_max reference values [57]. Therefore, we decided not to adhere to the standard VO_2_max classification thresholds, but rather based our comparison on values contained in our dataset, in effect comparing participants who expressed relatively higher and lower VO_2_max and average power values. Ultimately, two groups per parameter were established (relatively low power: < 8 W/kg and VO_2_max < 55 ml/kg/min, and relatively high power: > 8.5 W/kg, and VO_2_max > 62 ml/kg/min), further referred to as high and low power or high and low VO_2_max groups. Participants with intermediate values were not considered in the subsequent comparisons.

**Fig 7:**
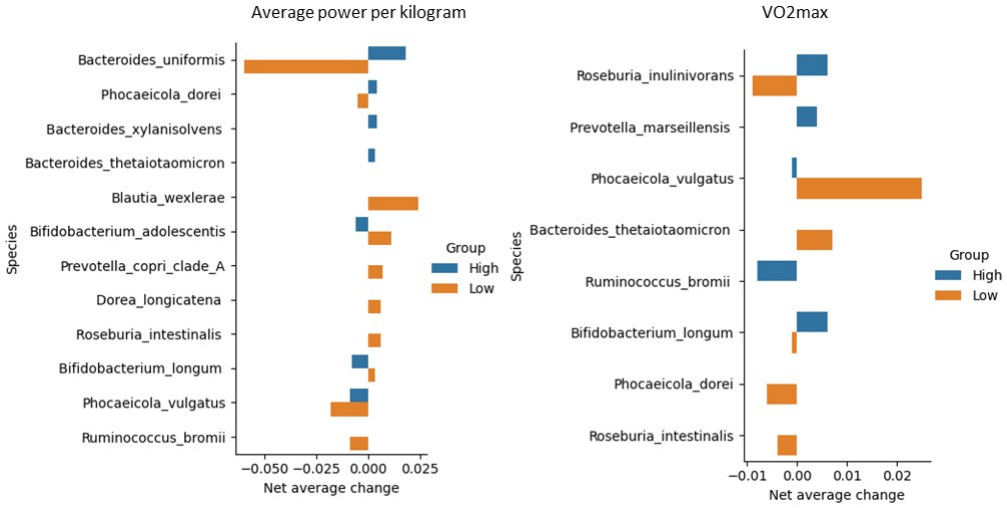
Species with the most significant differences in the average net abundance change between high and low power and VO_2_max groups.

For the high- and low-power groups, no alpha or beta diversity differences were seen. According to ANCOM, *Bifidobacterium longum* and *Bifidobacterium adolescentis* were the most differentially abundant species in the high group (CLR values above 0.4), whereas *Prevotella copri clade A* and *Ruminococcaceae unclassified* in the low group (CLR values above 0.3). However, these differences were not statistically significant. Comparison of high and low VO_2_max groups revealed subtle differences in alpha diversity (Fig 7, left), but no variation in beta diversity. *Firmicutes* to *Bacteroides* ratio, associated with VO_2_max [58], appeared to be more significant in some samples in the low versus high group (Fig 6, right). Differential abundance with ANCOM did not yield significant results but revealed species with the greatest differential enrichment. These included *Bidifobacterium longum*, *Candidatus Cibiobacter qucibialis* and *Bifidobacterium adolescentis* in the high VO_2_max group (CLR values above 0.5), and *Roseburia intestinalis*, *Prevotella copri clade A*, *Lachnospira pectinoschiza* in the low VO_2_max group (CLR values nearly 0.4 and above).

Longitudinal analyses identified the species with the most remarkable net average changes throughout the Bruce test. Fig 7 shows the top taxa with different high- and low-parameter group patterns. The most significant discrepancy was noted for *Bacteroides uniformis*, which drastically decreased in abundance in the low-power group and increased in the high-power group. *Bacteroides uniformis* facilitates glucose production and enhances physical performance [59]. *Roseburia inulinivorans* showed the highest sensitivity with regards to VO_2_max, with a slightly more significant decrease in the low group than in the high group. Like other species from the *Roseburia* genus, *Roseburia inulinivorans* is a short-chain fatty acid producer [60].

Interestingly, opposite trends were noted when comparing species across the high or low groupings according to the power and VO_2_max categories. *Bifidobacterium longum* increased in the high VO_2_max group and decreased in the low VO_2_max group, whereas a reversed pattern was seen in the corresponding power categories. *Phocaeicola vulgatus* decreased in abundance in both high groups and increased in VO_2_max, but decreased in average power. Finally, while no longitudinal changes emerged in the high groups regarding *Roseburia intestinalis* abundance, it was enriched in the low-power group and became less abundant in the low VO_2_max group.

**Fig 8:**
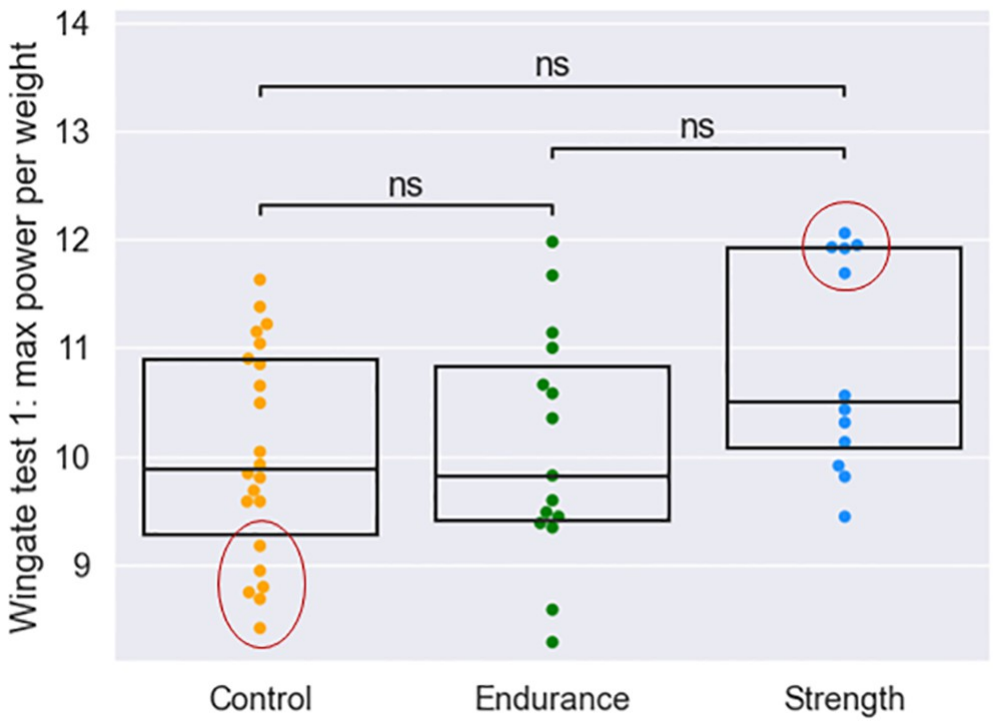
Distributions of maximum power values in strength, control and endurance groups, with marked high-power strength and low-power control individuals.

Finally, the bimodal distribution identified within groups in the maximum strength per kg plot (Fig 5) allowed us to fully differentiate between individuals who differ in training status and accompanying physiological characteristics. As a result, although small, two contrasting groups were established: strength individuals with visibly higher maximum average strength than the rest of the group and lesser trained individuals from the control group (Fig 8).

No alpha or beta diversity differences between the groups were identified. In terms of differential abundance with ANCOM, although no significant results were identified, there were substantially more abundant species in these groups. The species most enriched in the low-power control group were *Ruminococcus bicirculans*, *Bacteroides ovatus*, *Phocaeicola massiliensis* and *Bacteroides caccae* (CLR values above 0.5). In the high-power strength group, *Lachnospira eligens*, *Faecalibacterium prausnitzii*, *Bacteroides stercoris*, *Bifidobacterium pseudocatenulatum* and *Phocaeicola plebeius* were more enriched (CLR values above 0.5). Longitudinal analysis of the WT revealed a significant increase in the abundance of *Dysosmobacter welbionis* in the high group (average net change of 0.001) and a decrease in this species in the low group (average net change, 0.001). *Dysosmobacter welbionis* is found in approximately 65% of healthy individuals and is negatively correlated with TBMI and fasting blood glucose levels in obese individuals with metabolic syndrome [61]. No other volatile features were seen in the high-power group. The most significant increase in the low group was observed for *Bifidobacterium adolescentis* and a decrease in *Eubacterium rectale* (average net changes of 0.018 and –0.041, respectively).

## Discussion

Recent studies highlight that physical exercise positively influences the diversity and composition of the gut microbiome [62, 63]. Moreover, a higher maximal oxygen uptake, indicative of better cardiovascular fitness, is positively correlated with microbial diversity, which can be especially noticeable when comparing sedentary individuals with elite athletes, for whom this value can be twice as high [64]. To our knowledge, no study has yet compared the microbiome of trained (non-elite) and untrained individuals.

In our study, we compared microbiome alterations in strength and endurance athletes, as well as a control group, across two acute exercise bouts that engaged different energetic systems (WT and Bruce Treadmill Test), and identified sport-level and training-level microbiome characteristics. We discovered similar microbiome features across all participants, which was not surprising considering that all individuals were healthy, fit, and physically active. However, the differences identified herein, especially those linked to specific aspects of performance, have previously been found in elite athletes. We were particularly excited to see an enrichment of indigestible carbohydrate degrading bacteria in the endurance group, thereby suggesting performance-specific adaptations. Furthermore, a number of probiotic taxa with yet poorly defined mechanisms of action were found in the trained, and not in the non-trained, individuals.

Here we demonstrated a positive influence of physical fitness on microbial diversity and its correspondence with VO_2_max, but no differences emerged between the control, strength and endurance groups—probably because they were all similarly active, but not experiencing (or adapting to) more intense and diverse physical stressors as is the case in elite or professional athletes. This confirms that physical activity may be a hallmark of microbiome diversity, which correlates with general health and homeostasis maintenance [65].

Several species were consistently identified throughout our analyses. We were particularly interested in *Bifidobacterium longum* and *Bifidobacterium adolescentis*, as both correlated positively with all fitness parameters, most strongly withVO_2_max, and an increase in abundance in the high VO_2_max group throughout the Bruce Test. These two probiotic species are well represented in commercial products [66]. However, the effects of probiotic products on athletic performance are still poorly understood [67]. While we did not determine strain-level taxonomy from our samples [68], it was interesting that our results support the beneficial effects of *Bifidobacterium longum* and *Bifidobacterium adolescentis* on general fitness and cardiorespiratory fitness.

In contrast, we noted negative correlations between *Bacteroides* species and VO_2_max. Although *Bacteroides* are generally assumed to be present in a healthy microbiome [69], the *Firmicutes* to *Bacteroidetes* ratio was linked to a high VO_2_max [58]. We did not observe significant correlations between the *Firmicutes* to *Bacteroides* ratio and VO_2_max. On the other hand, we discovered a positive correlation between *Prevotella* and VO_2_max, and a high *Prevotella* to *Bacteroides* ratio is known to be associated with improved glucose metabolism [70] and increased glycogen storage. A high *Prevotella* to *Bacteroides* ratio was also seen in top Polish endurance athletes [71], but did not differ among e-sport players and physical education students [66].

We also found positive correlations between common SCFA producers (*Blautia wexlerae*, *Eubacterium rectale* and *Intestinimonas timonensis*) and maximal power during the WT. SCFAs can be used as an additional substrate for metabolism [72], being a desirable feature for endurance sports and related training. However, butyrate also induces beneficial alterations in skeletal tissues [73], which could explain the correlation between butyrate producers and power output.

The limitations of this study include the small sample size and missing W0 data. A larger sample would improve any inferences made, reduce bias, and could potentially identify individual responders (or non-responders) within a group. Expanding this analysis to include elite-level athletes would also be beneficial and allow a detailed examination of sport-specific microbiome associations among those well adapted to repeated aerobic or anaerobic oriented efforts. Of course, nutritional factors and dietary requirements (the proportion of proteins, fatty acids, carbohydrates, vitamins, and specific supplements) among elite sports people may further affect microbiome homeostasis. As already discussed, dietary intake can influence daily microbial shifts, as others have demonstrated [74, 75].

While transient microbiome shifts in response to dietary adjustments are well-documented, even within a 24h period, our design sought to address this confounder by providing a consistent dietary regimen at least two weeks before exercise testing. Moreover, participants’ diets were tailored to their energy needs, including an isocaloric balance. This means that they were not required to reduce their calorie intake, but were able to maintain their usual physical activities and training routines throughout this study. As such, we aimed to capture exercise-induced microbial variations independent of short-term dietary perturbations. Nevertheless, more accurate, long-term dietary tracking should be adopted in future studies to better estimate the impact of individual dietary factors.

It is worth emphasizing that the emerging physiological and pathophysiological roles of the intestinal human microbiome, both in terms of human health and sporting performance, are mainly due to the development of, and broader access to, molecular biology techniques. The development of molecular techniques, such as direct hybridization, now offers the possibility to obtain results that bypass the amplification procedure [76]. In the future, the incorporation of additional microbiome monitoring approaches, such as direct hybridization through experimental methods (DX hybridization) or metabolomics, should be considered. This complementary approach could further enhance our understanding of microbiome composition and function, offering valuable insight regarding their role in human health, performance and disease.

In summary, we demonstrated that fit and healthy individuals, regardless of their training level, displayed similar features to a healthy microbiome. Various individual responses were however observed, suggesting complex microbiota interactions or confounding factors. These observations underscore the intricate relationship between different forms of physical activity and expression of gut microbiota. Including more extreme cases, such as obese sedentary or elite athletes, would allow for better distinction between health-related and athlete-related microbiome features. Inclusion of additional data, such as metabolomics, would also provide deeper insight into individual baseline differences and interventional response (at any tissue level). The proposed approaches could move the field closer to explaining ambiguities of the current findings and help reveal the global role of the microbiome in physically active populations.

## Supporting information

S1 Fig Distributions of fitness parameter values per group with marked enterotypes. Green: *Bacteroides*-dominant, Blue: *Prevotella*-dominant, Orange: *Ruminococcus*-dominant.
(TIF)

S1 Table Alpha diversity results for control, endurance and strength groups at baseline. (DOCX)

S2 Table Beta diversity results for control, endurance and strength groups at baseline. (DOCX)

S3 Table Species and functions unique to control, endurance and strength. (DOCX)

S4 Table Top correlations of species with fitness parameter values calculated by SparCC. Only correlations above 0.1 or top correlations per fitness parameter if none above 0.1 included.
(DOCX)

S5 Table Correlations of functions with fitness parameter values calculated by SparCC. Only correlations above 0.1 or top correlations per fitness parameter if none above 0.1 included.
(DOCX)
