# Supplementary figures and images for: Microbiome features associated with performance measures in athletic and non-athletic individuals: A case-control study
Source: PLoS One. 2024 Feb 21;19(2):e0297858. doi: 10.1371/journal.pone.0297858 (PMC10880968; doi:10.1371/journal.pone.0297858)

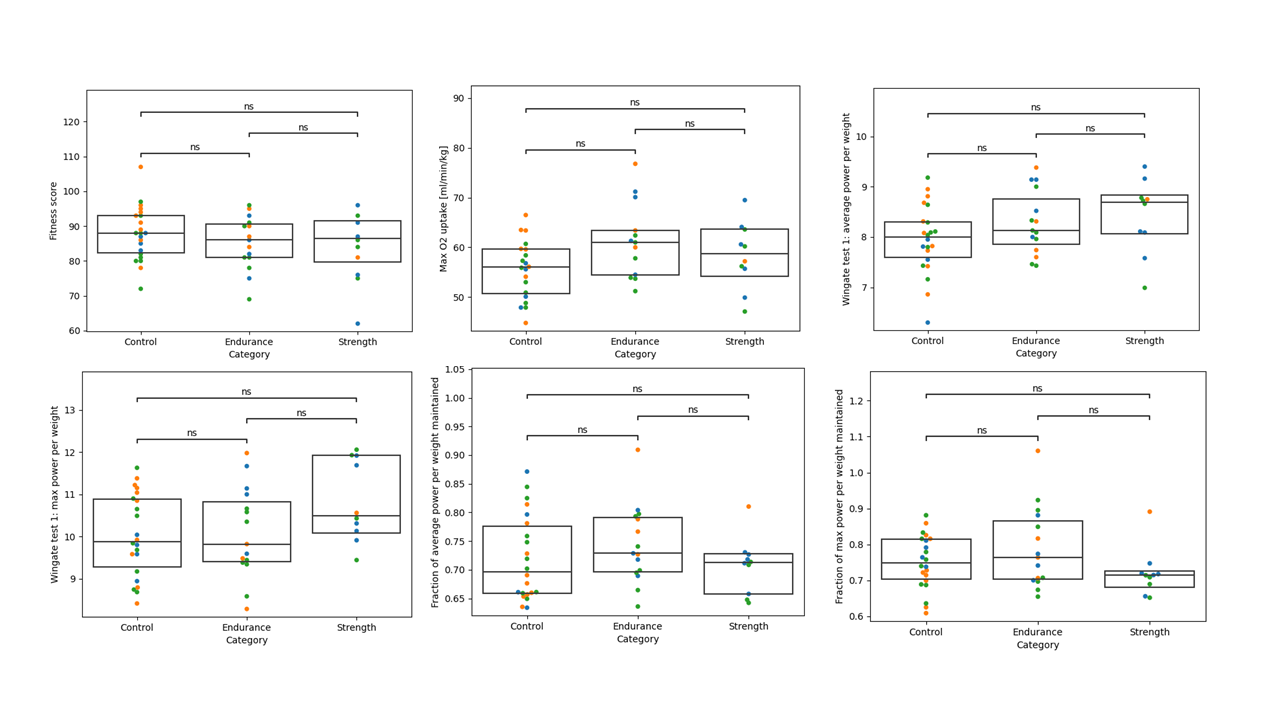

Supplement: S1 Fig — Green: Bacteroides-dominant, Blue: Prevotella-dominant, Orange: Ruminococcus-dominant. (TIF) [file pone.0297858.s001.tif]
