# Supplementary material for: Microbiome features associated with performance measures in athletic and non-athletic individuals: A case-control study
Source: PLoS One. 2024 Feb 21;19(2):e0297858. doi: 10.1371/journal.pone.0297858 (PMC10880968; doi:10.1371/journal.pone.0297858)
Supplement: S1 Table — (DOCX) [file pone.0297858.s002.docx]

| **Shannon entropy** | | | | |
| --- | --- | --- | --- | --- |
| **Timepoint** | **Groups** | **Kruskal-Wallis H** | **p-value** | **q-value** |
| W1 | Control vs Strength | 0.33 | 0.57 | 0.57 |
|  | Control vs Endurance | 2.22 | 0.14 | 0.41 |
|  | Strength vs Endurance | 0.48 | 0.49 | 0.57 |
| W2 | Control vs Strength | 0.01 | 0.92 | 0.92 |
|  | Control vs Endurance | 0.19 | 0.67 | 0.92 |
|  | Strength vs Endurance | 0.07 | 0.79 | 0.92 |
| B0 | Control vs Strength | 0.28 | 0.60 | 0.76 |
|  | Control vs Endurance | 0.12 | 0.73 | 0.76 |
|  | Strength vs Endurance | 0.10 | 0.76 | 0.76 |
| B1 | Control vs Strength | 0.04 | 0.85 | 0.85 |
|  | Control vs Endurance | 0.56 | 0.46 | 0.68 |
|  | Strength vs Endurance | 0.57 | 0.45 | 0.68 |
| B2 | Control vs Strength | 0.78 | 0.38 | 0.61 |
|  | Control vs Endurance | 0.70 | 0.40 | 0.61 |
|  | Strength vs Endurance | 0.00 | 0.95 | 0.95 |
| **Simpson** | | | | |
| **Timepoint** | **Groups** | **Kruskal-Wallis H** | **p-value** | **q-value** |
| W1 | Control vs Strength | 0.43 | 0.51 | 0.77 |
|  | Control vs Endurance | 0.02 | 0.90 | 0.90 |
|  | Strength vs Endurance | 0.63 | 0.43 | 0.77 |
| W2 | Control vs Strength | 0.56 | 0.45 | 0.45 |
|  | Control vs Endurance | 2.22 | 0.14 | 0.31 |
|  | Strength vs Endurance | 1.51 | 0.22 | 0.33 |
| B0 | Control vs Strength | 0.80 | 0.37 | 0.55 |
|  | Control vs Endurance | 0.00 | 0.98 | 0.98 |
|  | Strength vs Endurance | 1.50 | 0.22 | 0.55 |
| B1 | Control vs Strength | 0.00 | 0.95 | 0.95 |
|  | Control vs Endurance | 0.66 | 0.42 | 0.65 |
|  | Strength vs Endurance | 0.61 | 0.44 | 0.65 |
| B2 | Control vs Strength | 0.26 | 0.61 | 0.61 |
|  | Control vs Endurance | 1.81 | 0.18 | 0.28 |
|  | Strength vs Endurance | 1.76 | 0.18 | 0.28 |
| **Observed features** | | | | |
| **Timepoint** | **Groups** | **Kruskal-Wallis H** | **p-value** | **q-value** |
| W1 | Control vs Strength | 0.43 | 0.51 | 0.51 |
|  | Control vs Endurance | 1.35 | 0.25 | 0.37 |
|  | Strength vs Endurance | 4.10 | 0.04 | 0.13 |
| W2 | Control vs Strength | 0.56 | 0.45 | 0.45 |
|  | Control vs Endurance | 2.22 | 0.14 | 0.31 |
|  | Strength vs Endurance | 1.51 | 0.22 | 0.33 |
| B0 | Control vs Strength | 1.07 | 0.30 | 0.72 |
|  | Control vs Endurance | 0.13 | 0.72 | 0.72 |
|  | Strength vs Endurance | 0.23 | 0.63 | 0.72 |
| B1 | Control vs Strength | 1.11 | 0.29 | 0.54 |
|  | Control vs Endurance | 0.37 | 0.54 | 0.54 |
|  | Strength vs Endurance | 0.78 | 0.38 | 0.54 |
| B2 | Control vs Strength | 0.17 | 0.68 | 0.68 |
|  | Control vs Endurance | 0.17 | 0.68 | 0.68 |
|  | Strength vs Endurance | 0.85 | 0.36 | 0.68 |

B0: in the morning fasting before the Bruce Trademill Test, B1: on the same day after the Bruce Trademill Test, B2: morning fasting after the Bruce Trademill Test on an empty stomach, W1: the same day after the Wingate Anaerobic Test, W2: morning fasting after the WAnT on an empty stomach
