# Supplementary material for: Microbiome features associated with performance measures in athletic and non-athletic individuals: A case-control study
Source: PLoS One. 2024 Feb 21;19(2):e0297858. doi: 10.1371/journal.pone.0297858 (PMC10880968; doi:10.1371/journal.pone.0297858)
Supplement: S2 Table — (DOCX) [file pone.0297858.s003.docx]

| **Bray-Curtis** | | | | |
| --- | --- | --- | --- | --- |
| **Timepoint** | **Groups** | **Permanova pseudo-F** | **p-value** | **q-value** |
| W1 | Control vs Strength | 0.74 | 0.73 | 0.73 |
|  | Control vs Endurance | 0.79 | 0.69 | 0.73 |
|  | Strength vs Endurance | 1.40 | 0.14 | 0.42 |
| W2 | Control vs Strength | 0.62 | 0.89 | 0.89 |
|  | Control vs Endurance | 1.58 | 0.07 | 0.11 |
|  | Strength vs Endurance | 1.90 | 0.03 | 0.11 |
| B0 | Control vs Strength | 0.86 | 0.59 | 0.77 |
|  | Control vs Endurance | 0.71 | 0.77 | 0.77 |
|  | Strength vs Endurance | 1.28 | 0.22 | 0.65 |
| B1 | Control vs Strength | 0.83 | 0.65 | 0.65 |
|  | Control vs Endurance | 1.08 | 0.33 | 0.50 |
|  | Strength vs Endurance | 1.16 | 0.27 | 0.50 |
| B2 | Control vs Strength | 0.62 | 0.87 | 0.87 |
|  | Control vs Endurance | 0.65 | 0.85 | 0.87 |
|  | Strength vs Endurance | 0.78 | 0.65 | 0.87 |
| **Jaccard** | | | | |
| **Timepoint** | **Groups** | **Permanova pseudo-F** | **p-value** | **q-value** |
| W1 | Control vs Strength | 0.73 | 0.93 | 0.93 |
|  | Control vs Endurance | 1.00 | 0.41 | 0.62 |
|  | Strength vs Endurance | 1.27 | 0.09 | 0.28 |
| W2 | Control vs Strength | 0.84 | 0.74 | 0.76 |
|  | Control vs Endurance | 0.93 | 0.60 | 0.76 |
|  | Strength vs Endurance | 0.84 | 0.76 | 0.76 |
| B0 | Control vs Strength | 0.80 | 0.84 | 0.84 |
|  | Control vs Endurance | 0.95 | 0.51 | 0.84 |
|  | Strength vs Endurance | 0.82 | 0.81 | 0.84 |
| B1 | Control vs Strength | 1.02 | 0.37 | 0.55 |
|  | Control vs Endurance | 1.03 | 0.33 | 0.55 |
|  | Strength vs Endurance | 0.86 | 0.72 | 0.72 |
| B2 | Control vs Strength | 0.92 | 0.55 | 0.61 |
|  | Control vs Endurance | 0.90 | 0.61 | 0.61 |
|  | Strength vs Endurance | 0.96 | 0.46 | 0.61 |

B0: in the morning fasting before the Bruce Trademill Test, B1: on the same day after the Bruce Trademill Test, B2: morning fasting after the Bruce Trademill Test on an empty stomach, W1: the same day after the Wingate Anaerobic Test, W2: morning fasting after the WAnT on an empty stomach
