# Supplementary material for: Microbiome features associated with performance measures in athletic and non-athletic individuals: A case-control study
Source: PLoS One. 2024 Feb 21;19(2):e0297858. doi: 10.1371/journal.pone.0297858 (PMC10880968; doi:10.1371/journal.pone.0297858)
Supplement: S4 Table — Only correlations above 0.1 or top correlations per fitness parameter if none above 0.1 included. (DOCX) [file pone.0297858.s005.docx]

| - **Fitness parameter** | - **Species** | - **r** |
| --- | --- | --- |
| - Fitness score | - *Phocaeicola vulgatus* | - 0.17 |
| - Fitness score | - *Bacteroides eggerthii* | - 0.11 |
| - Fitness score | - *Streptococcus thermophilus* | - 0.11 |
| - Fitness score | - *GGB3293 SGB4348* | - -0.19 |
| - Fitness score | - *Roseburia intestinalis* | - -0.17 |
| - VO_2_max | - *Bifidobacterium adolescentis* | - 0.18 |
| - VO_2_max | - *Bifidobacterium longum* | - 0.14 |
| - VO_2_max | - *Candidatus Cibiobacter qucibialis* | - 0.11 |
| - VO_2_max | - *Phocaeicola massiliensis* | - -0.13 |
| - VO_2_max | - *Lachnospira pectinoschiza* | - -0.13 |
| - VO_2_max | - *Eubacterium siraeum* | - -0.13 |
| - VO_2_max | - *Eubacterium siraeum* | - -0.13 |
| - Average power | - *Clostridiaceae bacterium* | - 0.09 |
| - Average power | - *Ruminococcus bromii* | - 0.08 |
| - Average power | - *Blautia wexlerae* | - 0.07 |
| - Average power | - *Clostridium innocuum* | - -0.08 |
| - Average power | - *GGB1227 SGB1600* | - -0.08 |
| - Average power | - *Roseburia inulinivorans* | - -0.07 |
| - Maximal power | - *Eubacterium rectale* | - 0.09 |
| - Maximal power | - *Blautia wexlerae* | - 0.09 |
| - Maximal power | - *GGB38171 SGB72433* | - 0.08 |
| - Maximal power | - *Phocaeicola massiliensis* | - -0.08 |
| - Maximal power | - *GGB9627 SGB15081* | - -0.07 |
| - Maximal power | - *Prevotella buccalis* | - -0.07 |
