# Supplementary material for: Microbiome features associated with performance measures in athletic and non-athletic individuals: A case-control study
Source: PLoS One. 2024 Feb 21;19(2):e0297858. doi: 10.1371/journal.pone.0297858 (PMC10880968; doi:10.1371/journal.pone.0297858)
Supplement: S5 Table — Only correlations above 0.1 or top correlations per fitness parameter if none above 0.1 included. (DOCX) [file pone.0297858.s006.docx]

| - **Fitness parameter** | - **Function** | - **Species associated** | - **r** |
| --- | --- | --- | --- |
| - Fitness score | - PWY-7238: - sucrose biosynthesis II | - *Bifidobacterium adolescentis* | - 0.06 |
| - Fitness score | - PWY-1042: - glycolysis IV | - *Bacteroides vulgatus* | - -0.06 |
| - Fitness score | - PANTO-PWY: - phosphopantothenate biosynthesis I | - *Ruminococcus torques* | - -0.07 |
| - Fitness score | - PWY-5695: - inosine 5'-phosphate degradation | - *Fusicatenibacter saccharivorans* | - -0.07 |
| - VO_2_max | - PWY-6277: - superpathway of 5-aminoimidazole ribonucleotide biosynthesis | - *Blautia obeum* | - -0.06 |
| - VO_2_max | - PWY-8178: - pentose phosphate pathway (non-oxidative branch) II | - *Fusicatenibacter saccharivorans* | - 0.06 |
| - VO_2_max | - PWY-6317: - D-galactose degradation I (Leloir pathway | - *Faecalibacterium prausnitzii* | - 0.06 |
| - VO_2_max | - NONOXIPENT-PWY: - pentose phosphate pathway (non-oxidative branch) I | - *Fusicatenibacter saccharivorans* | - 0.06 |
| - VO_2_max | - PWY-7357: - thiamine phosphate formation from pyrithiamine and oxythiamine (yeast) | - *Blautia obeum* | - -0.06 |
| - VO_2_max | - PWY-5103: - L-isoleucine biosynthesis III | - *Prevotella copri* | - -0.07 |
| - VO_2_max | - PWY-7237: - myo-, chiro- and scyllo-inositol degradation | - *Ruminococcus torques* | - 0.07 |
| - VO_2_max | - PWY-6823: - molybdopterin biosynthesis | - *Blautia obeum* | - -0.07 |
| - VO_2_max | - PWY-6147: - 6-hydroxymethyl-dihydropterin diphosphate biosynthesis I | - *Prevotella copri* | - 0.07 |
| - VO_2_max | - LACTOSECAT-PWY: - lactose and galactose degradation I | - *Collinsella aerofaciens* | - -0.07 |
| - VO_2_max | - ARO-PWY: - chorismate biosynthesis I | - *Faecalibacterium prausnitzii* | - -0.09 |
| - VO_2_max | - PWY0-1296: - purine ribonucleosides degradation | - *Fusicatenibacter saccharivorans* | - -0.11 |
| - Average power | - PWY-6387: - UDP-N-acetylmuramoyl-pentapeptide biosynthesis I (meso-diaminopimelate containing) | - *Faecalibacterium prausnitzii* | - -0.06 |
| - Average power | - ARGSYN-PWY: - L-arginine biosynthesis I (via L-ornithine) | - *Blautia wexlerae* | - 0.07 |
| - Average power | - PWY-7238: - sucrose biosynthesis II | - *Blautia wexlerae* | - -0.08 |
| - Maximum power | - PWY66-429: fatty acid biosynthesis initiation (mitochondria) | - *Fusicatenibacter saccharivorans* | - -0.09 |
| - Maximum power | - PWY-6823: molybdopterin biosynthesis | - *Blautia obeum* | - -0.11 |
